# The Transactions of the American Medical Association

**Published:** 1850-02

**Authors:** 


ART. VI.—*The Transactions of the American Medical Association.* Instituted 1847. Vol. II. Philadelphia: Printed for the Association, by T. K. & P. G. Collins. 1849.

This ponderous tome, consisting of nearly one thousand pages, has reached us at a time when we have but little space for doing much justice to its merits. Its contents are of no ordinary value to the American physician, for they are made up of matter that concerns his practical duties, and are contributed by physicians who are scattered over the various regions of this Confederacy, and who are thus enabled to pour out the treasures of experience from every quarter of the Union. The transactions of this association are so interesting, that we feel it to be our duty to present our readers with a copy of the table of contents, so far as to embrace the practical material of the work. The following reports were submitted:

A.—*Report of the Committee on Medical Sciences.*

B.—*Report of the Committee on Practical Medicine.* 1. On the more prevalent diseases of Swedsboro', New Jersey, and its neighborhood, during the year 1848. By J. F. Garrison, M. D. 2. An account of the dysentery, as it prevailed in Cambridge, Massachusetts, in the years 1847 and 1848. By Worrill Wyman, M. D. 3. On bilious fever, as it prevails in the eastern portions of New Jersey. By J. Fithian, M. D., of Woodbury, New Jersey. 4. On erysipelas, as it prevailed in the eastern portion of New Jersey; with some remarks on erysipelas of the respiratory mucous membrane. By J. Fithian, M. D., of Woodbury, New Jersey.

C.—*Report of the Committee on Surgery.*

D.—*Report of the Committee on Obstetrics.* 1. Case of retroflexion of the uterus. By A. C. Post, M. D., of New

York. 2. Case of the same. By B. W. McCready, M. D., of New York.

E.—*Report of the Committee on Medical Education.* 1. The views of the medical faculty of Harvard University relative to the extension of the lecture term. 2. Report of the special committee appointed to prepare " a statement of the facts and arguments which may be adduced in favor of the prolongation of the courses of medical lectures to six months."

F.—*Report of the Committee on Medical Literature.*

G.—*First Report of the Committee on Public Hygiene of the American Medical Association.* Report on the sanitary condition of Concord, New Hampshire. Report on the sanitary condition of Portland, Maine. Hygiene of New York city. Sanitary condition of Philadelphia. Public hygiene of Massachusetts; particularly of the cities of Boston and Lowell. Sanitary condition of Baltimore; Charleston, South Carolina; New Orleans, Louisiana; Louisville, Kentucky; and of Cincinnati. The yellow fever quarantine at New Orleans. On the influence upon health of the introduction of tea and coffee in large proportion into the dietary of children and the laboring classes. By S. Jackson, M. D. On the introduction of water and gas into cities. Letter from the Chief of the Bureau of Medicine and Surgery, Navy Department, on the use of disinfectants in the navy.

H.—*Report on Adulterated and Sophisticated Drugs.*

I.—*Report on Indigenous Medical Botany.* On indigenous medicinal plants of South Carolina. On the indigenous medicinal botany of Massachusetts.

We have thus given a glimpse of the character of the labors of the American Medical Association. The reader cannot fail to see from the above table, the extent, scope and design of the annual meeting of this association, and he may reasonably infer that the labors of such a body are of a character to elevate, improve and advance the medical profession. It is the bounden duty of every physician, who feels any interest in these things, to contribute towards the success of the association, and to labor to perpetuate the existence of this national congregation of physicians.

Those who are desirous of obtaining copies of the volumes published by the association can do so on the following terms: The second volume, the one before us, is furnished in paper covers, at $3 per copy, and in muslin binding at $3 50. Societies which have been represented in the association, will be furnished copies for their members on the following terms: three copies of volume II, in paper covers, for $5 00; three copies of volumes I and II, for $8 00. The amount must be remitted to Isaac Hays, M. D., Philadelphia, treasurer of the association, in par funds.

The next meeting of the American Medical Association will take place in May, 1850, in Cincinnati, Ohio, and we hope the West and South will be largely represented on the occasion.

We are indebted to the courtesy of Messrs. Beckwith & Morton for copies of the various works we have noticed in the present number.

